# Variability in disease severity among cystic fibrosis patients carrying residual-function variants: data from the European Cystic Fibrosis Society Patient Registry

**DOI:** 10.1183/23120541.00587-2024

**Published:** 2025-01-13

**Authors:** Meir Mei-Zahav, Annalisa Orenti, Andreas Jung, Eitan Kerem

**Affiliations:** 1 Kathy and Lee Graub Cystic Fibrosis Center and Pulmonary Unit, Schneider Children's Medical Center of Israel, Petah Tikva, Israel; 2 Faculty of Medicine, Tel Aviv University, Tel Aviv, Israel; 3 Department of Clinical Sciences and Community Health, Laboratory of Medical Statistics, Biometry and Epidemiology “G.A. Maccacaro”, University of Milan, Milan, Italy; 4 Department of Paediatric Pulmonology, University Children's Hospital Zurich, Zurich, Switzerland; 5 Department of Pediatrics and Center for Cystic Fibrosis, Hadassah University Medical Center, Hebrew University Hadassah Medical School, Jerusalem, Israel

## Abstract

**Background:**

People with cystic fibrosis (CF) variants that exhibit residual function (RF) of the CF transmembrane conductance regulator are considered to have a milder disease; however, the spectrum of CF phenotype within the different RF variants has not been extensively investigated. The aim of the present study was to characterise the spectrum of CF disease severity in people with CF (pwCF) carrying different RF variants, using the European Cystic Fibrosis Society Patient Registry (ECFSPR) data.

**Methods:**

A retrospective cross-sectional and longitudinal cohort study included data from the ECFSPR during 2008–2016. Demographic and clinical characteristics of pwCF carrying different RF variants were compared with the characteristics of pwCF who are homozygous for F508del. Among those with RF, a distinction was made between pwCF carrying class IV or class V variants and pwCF carrying specific RF variants.

**Results:**

Out of 56 701 pwCF in the ECFSPR, 6192 carried RF variants and 22 766 were homozygous for F508del. Class IV/F508del variants were associated with a milder course than class V/F508del; both were milder than pwCF homozygous for F508del. Forced expiratory volume in 1 s % predicted (FEV_1_pp) declined in childhood in all groups. For adults, the hazard ratio of death for class V/F508del *versus* class IV/F508del was 2.14 (95% confidence interval 0.99–4.63, p=0.052). PwCF carrying 3849+10 kb C→T/F508del and pwCF carrying R334W/F508del had age-specific FEV_1_pp and chronic bacterial colonisation similar to those of pwCF homozygous for F508del.

**Conclusion:**

There is a wide spectrum of disease severity between the different RF variants. Some, such as those carrying 3849+10 kb C→T, have severe disease, similar to that of pwCF homozygous for F508del.

## Shareable abstract

**Cystic fibrosis patients carrying residual-function mutations exhibit diverse pulmonary disease. Certain residual-function mutations result in a disease that is not different from the disease of patients carrying two F508del mutations.** https://bit.ly/3A3ydJA

## Introduction

Cystic fibrosis (CF) is a life-shortening disease caused by variants in the cystic fibrosis transmembrane conductance regulator (CFTR) gene [1, 2]. Despite being classified as a monogenic disorder, CF exhibits a wide range of disease. Extensive genotype–phenotype studies have unveiled the intricate landscape of CFTR variants and their diverse effects on disease manifestation expressions [3–5]. Notably, approximately 15% of the CF population carries variants classified as exhibiting residual CFTR ion transport, known as “residual function” (RF) variants. The variants among this unique subgroup allow for some degree of CFTR protein activity, albeit reduced, providing a captivating glimpse into the intricate interplay between genotype and phenotype in CF [6, 7]. By contrast, variants associated with minimal or no CFTR function are termed “minimal function” (MF) variants.

People with CF (pwCF) who carry RF variants often experience a milder form of the disease. They are more likely to retain sufficient pancreatic function, ensuring normal nutrition, and their sweat chloride values may fall within the normal, borderline or slightly elevated range, indicating partially preserved CFTR activity. In a previous study encompassing 44 594 individuals listed in the European Cystic Fibrosis Society Patient Registry (ECFSPR) [6], we demonstrated that patients carrying RF variants tended to be older at diagnosis, had lower sweat chloride levels upon diagnosis and exhibited higher pulmonary function (forced expiratory volume in 1 s % predicted (FEV_1_pp)) across various age groups. However, their FEV_1_pp values declined with age, and a significant proportion of RF patients eventually showed FEV_1_pp values comparable to those of patients with MF variants within the same age groups. Furthermore, individuals with RF variants had shorter lifespans than the non-CF population, with mortality becoming apparent around the age of 20 years [6].

Given the rarity of RF variants, previous studies focusing on specific RF variants have been constrained by small sample sizes of pwCF. Salvatore *et al*. [7], in their analysis of Italian pwCF, hinted at clinical heterogeneity among different RF variants. However, comprehensive data on large cohorts of pwCF carrying specific RF variants are needed. In this article, we embark on a comprehensive analysis of disease severity among pwCF, with a particular focus on individuals harbouring RF CFTR variants. Leveraging meticulously gathered data from the ECFSPR [8], which comprises a large, diverse and extensive CF population, we aim to illuminate the disparities in clinical outcomes observed within this subgroup. This invaluable dataset empowers us to delve into the nuanced impact of RF variants on lung function, nutritional status, infection prevalence and more.

The utilisation of the ECFSPR allowed us to conduct a rigorous comparison of disease severity between pwCF carrying at least one RF variant and those carrying two MF variants. This endeavour brings us one step closer to personalised approaches in diagnosis, treatment, genetic counselling and therapeutic interventions tailored to an individual's unique CFTR variant profile.

## Methods

### Data source

The ECFSPR serves as our data repository. It diligently accumulates demographic and clinical data from consenting individuals with CF across Europe. These data are collected in accordance with predefined inclusion criteria and definitions, allowing us to measure, survey and compare various clinical aspects of CF and its treatments in European countries. As of our study's initiation, the registry encompasses information from >54 000 pwCF hailing from 40 participating countries, offering a longitudinal view spanning from 2008 to 2021.

### Study design

We conducted a retrospective cross-sectional cohort study and a longitudinal study on a subgroup of individuals with two primary objectives: (1) to assess the disease severity of pwCF carrying at least one RF variant and (2) to analyse and compare the clinical presentations of specific RF variants. We included all pwCF listed in the ECFSPR for any year between 2008 and 2016 who met our predefined inclusion criteria. To minimise the potential bias introduced by the use of CFTR modulators, we collected data up to 2016, as these medications were not widely employed until 2017, with the exception of ivacaftor for class III variants, which were not considered in our study.

### Data collection

The ECFSPR collects demographic and clinical variables from pwCF, including age; age at diagnosis; sex; country of origin; history of lung and liver transplants; sweat-test results; presence of meconium ileus; FEV_1_pp values computed using Global Lung Function Initiative (GLI) equations; annual percent change in FEV_1_pp; body mass index (BMI); BMI z-scores; presence of chronic *Staphylococcus aureus*; chronic *Pseudomonas aeruginosa* (PSA); chronic *Burkholderia cepacia* complex; *Stenotrophomonas maltophilia*; nontuberculous mycobacteria; allergic bronchopulmonary aspergillosis; CF-related diabetes (CFRD) categorised by insulin use; instances of major haemoptysis exceeding 250 mL; pneumothorax occurrences; liver disease; and various treatments including hypertonic saline, DNase, inhaled antibiotics, azithromycin, bronchodilators, oxygen therapy, pancreatic enzyme supplementation (as a surrogate for pancreatic function) and ursodeoxycholic acid. The ECFSPR does not collect data on an individual's socioeconomic status. As a proxy of this, we used the relevant country's gross national income per capita, computed with the Atlas method, and we divided the countries into two groups: countries with a gross national income in 2016 of <USD 25 000 were considered to be lower income.

### Variant classification

In our previous study [6], all variants present in the ECFSPR dataset were categorised into classes I to V or marked as unknown (supplementary tables 1 and 2). This classification adhered to the CFTR2 definition [9] and was consistent with previous studies that classified CFTR variants [10]. We defined classes IV and V as RF variants; a comprehensive list of all RF variants is provided in supplementary tables 1 and 2. Our study included pwCF with RF/RF and MF/RF variants, with separate analyses conducted for class IV and class V variants. A further selection, to have a more homogeneous group of pwCF, was conducted on pwCF with F508del and one of the most common RF variants. PwCF who are homozygous for F508del variants always served as the control group.

**TABLE 1:** The most common residual-function (RF) variants in this study, representing 74% of all RF variants (4831/6516)

Variant name	Class	CFTR alleles, n (%)
R117H p.Arg117His, c.350G>A	IV	1047 (16.07)
2789+5G→A, p.?, c.2657+5G>A	V	940 (14.43)
3849+10 kb C→T,p.?,c.3718-2477C>T, or c.3717+12191C>T	V	913 (14.01)
D1152H, p.Asp1152His, c.3454G>C	IV	515 (7.90)
3272-26A→G, p.?, c.3140-26A>G	V	468 (7.18)
R334W, p.Arg334Trp, c.1000C>T	IV	393 (6.03)
A455E, p.Ala455Glu, c.1364C>A	IV	290 (4.45)
L206W, p.Leu206Trp, c.617T>G	IV	265 (4.07)

CFTR: cystic fibrosis transmembrane conductance regulator.

**TABLE 2:** Demographic and clinical characteristics of F508del homozygote and heterozygote people with cystic fibrosis (pwCF) carrying class IV or class V variants, p-values refer to the comparison with the F508del homozygote group

	F508del/F508del (N=22 766)	F508del/class IV (N=1788)	F508del/class IV *versus* F508del/F508del, p-value^#^	F508del/class V (N=1499)	F508del/class V *versus* F508del/F508del, p-value^#^	F508del/class IV *versus* F508del/class V, p-value^#^
**Demographic characteristics**						
Age, years, median (IQR)	19.6 (10.5–29.2)	24.3 (9.4–41.3)	<0.001	27.1 (15.5–41.5)	<0.001	<0.001
Female	10 867 (47.73)	886 (49.55)	0.140	737 (49.17)	0.286	0.834
Low-income country	2466 (10.83)	83 (4.64)	<0.001	230 (15.34)	<0.001	<0.001
Age at diagnosis, years, median (IQR)	0.2 (0.1–1.4)	5.3 (0.2–26.4)	<0.001	9.4 (0.7–20.5)	<0.001	0.006
**Diagnosis**						
Sweat chloride value (Cl, mEq·L^−1^), median (IQR)	100 (90–111)	57 (38–79)	<0.001	84.1 (67–100)	<0.001	<0.001
**Sputum pathogens**						
Chronic *Pseudomonas aeruginosa*	6716 (35.13)	243 (14.91)	<0.001	411 (31.59)	<0.001	<0.001
Chronic *Burkholderia cepacia complex*	728 (3.80)	21 (1.26)	<0.001	37 (2.84)	0.092	0.070
Chronic *Staphylococcus aureus*	5459 (36.45)	288 (23.72)	<0.001	424 (44.31)	0.011	<0.001
**Complications**						
ABPA	1487 (6.89)	52 (3.04)	<0.001	63 (4.46)	0.053	0.036
CF-related diabetes	4897 (22.33)	60 (3.50)	<0.001	59 (4.18)	<0.001	0.676
**Treatment**						
Continuous inhaled hypertonic NaCl	8256 (43.57)	348 (20.81)	<0.001	471 (35.76)	<0.001	<0.001
Continuous use of rhDNase	13 048 (59.20)	497 (28.38)	<0.001	715 (49.24)	<0.001	<0.001
Inhaled continuous antibiotic	11 015 (51.05)	355 (20.51)	<0.001	555 (38.7)	<0.001	<0.001
Continuous azithromycin	7499 (39.75)	326 (19.52)	<0.001	426 (32.44)	<0.001	<0.001
Continuous inhaled bronchodilators	12 500 (65.97)	721 (43.04)	<0.001	843 (63.91)	0.005	<0.001
Oxygen therapy	1857 (8.58)	62 (3.65)	<0.001	92 (6.42)	<0.001	0.015
Lung transplantation	1742 (7.78)	35 (1.97)	<0.001	72 (4.86)	<0.001	0.002
**FEV_1_pp, median (IQR)**						
6–11 years	94.5 (82.7–103.7)	99.2 (91.4–108.9)	<0.001	98.2 (89.8–108.2)	0.001	0.511
12–17 years	83.1 (68.4–94.9)	93.6 (85.3–102.3)	<0.001	92.3 (78.6–100.7)	<0.001	0.015
18–34 years	66.0 (45.5–83.1)	86.8 (70.4–97.9)	<0.001	75.2 (56.2–91.2)	<0.001	<0.001
35–49 years	55.0 (38.0–75.7)	79.9 (60.7–95.4)	<0.001	66.1 (45.5–85.1)	0.001	<0.001
50+ years	51.8 (34.2–71.3)	69.4 (49.9–90.2)	<0.001	59.6 (40.3–77.8)	0.087	<0.001

Data are presented as n (%) unless otherwise stated. IQR: interquartile range; ABPA: allergic bronchopulmonary aspergillosis; CF: cystic fibrosis; FEV_1_pp: forced expiratory volume in 1 s % predicted. ^#^: p-value was adjusted to age, sex, age at diagnosis and socioeconomic status, except for the demographic characteristics, for which unadjusted p-values are presented.

### Data analysis

Categorical variables were summarised using counts and percentages after excluding missing values, while numerical variables were described using medians and quartiles due to their asymmetric distributions. To compare characteristics between two groups, chi-squared tests were employed for categorical variables, and Wilcoxon tests were used for numerical variables. False discovery rate adjustments were applied to account for multiple comparisons. Linear and logistic regression models were fitted for numerical and categorical variables, respectively, adjusting for the genetic group and confounding factors such as age, sex, age at diagnosis and socioeconomic status. In this cross-sectional cohort analysis, for each subject, we used the most recent data available between 2008 and 2016.

### Sub-analysis

A sub-analysis was conducted for pwCF listed in the ECFSPR who met our inclusion criteria and possessed longitudinal FEV_1_pp data for at least 3 consecutive years before 2016. This sub-analysis aimed to assess the rate of FEV_1_pp decline in the different variant groups. We employed a mixed-effects regression model, treating FEV_1_pp as the response variable and variant group as the explanatory variable. A random effect for pwCF was included to account for within-patient correlations. All measurements available from 2008 to 2016 were utilised in this model. In addition, a multiple regression model was fitted to adjust for potential confounding factors, including sex, age, country income group, chronic PSA, CFRD and chronic *Burkholderia cepacia* complex.

### Survival analysis

To evaluate the prognostic role of specific variant groups, we conducted survival analysis. Kaplan-Meier non-parametric curves were generated for each variant group. Furthermore, a Cox regression model using age as the timescale and accounting for late entry was employed to assess the impact of variant group on the probability of mortality at a specific age. Each subject is considered at risk in the model from time of entry in the registry data till death or end of follow-up (31/12/2016). This analysis accounted for adjusting factors such as sex, age, country, chronic PSA, CFRD, and chronic *Burkholderia cepacia* complex.

All analyses and sub-analyses were done after excluding missing values. No imputation of missing values was done. Variables with >10% of values missing were excluded from the regression models.

### Ethics considerations

All participating centres and national registries obtained ethics approval and patients’ informed consent for data collection and ECFSPR participation, including consent for future research data usage. Our study received approval from the ECFSPR Scientific Committee and the ECFSPR Steering Committee.

## Results

**FIGURE 1:**
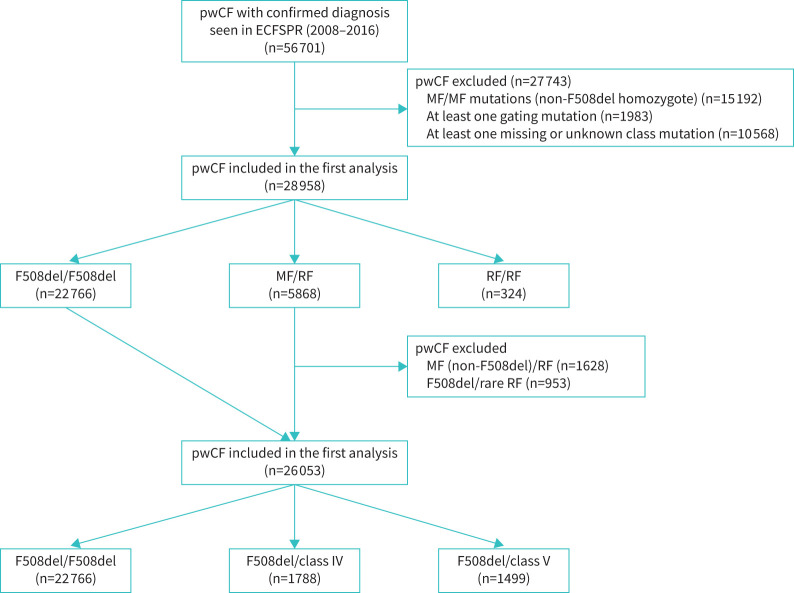
The people with cystic fibrosis (pwCF) included in the study. ECFSPR: European Cystic Fibrosis Society Patient Registry; MF: minimal function; RF: residual function.

The ECFSPR registered a total of 56 701 individuals with CF seen in the years 2008–2016. Among them, 15 192 individuals exhibiting MF/MF variants were excluded, except those who were homozygous for the F508del variant. In addition, 1983 with at least one gating variant were excluded and a further 10 568 individuals with CF who did not meet the inclusion criteria due to a lack of one RF variant were excluded (figure 1). The first step of the analysis comprised 6192 individuals with CF who had at least one RF variant, while the control group consisted of 22 766 individuals who were homozygous for the F508del variant (figure 1). The clinical characteristics of the RF group and a comparison between pwCF carrying two RF variants and pwCF who were compound heterozygous to RF/MF are presented in the supplementary material. There was no difference in most clinical parameters between the two groups (supplementary table 3).

**TABLE 3:** Results of the multiple Cox regression model on the adults, this analysis was performed only in adults as there is no difference in survival among children

Variables	Hazard ratio (95% CI)	p-value
**Variant group (F508del/class V *versus* F508del/class IV)**	2.14 (0.99–4.63)	0.052
**Variant group (F508del/F508del *versus* F508del/class IV)**	4.48 (2.08–9.65)	<0.001
**Sex (female *versus* male)**	1.32 (1.19–1.47)	<0.001
**Chronic *Pseudomonas aeruginosa* (yes *versus* no)**	1.27 (0.87–1.86)	0.220
**Chronic *Burkholderia cepacia* complex (yes *versus* no)**	2.69 (2.26–3.19)	<0.001
**Diabetes insulin treated (yes *versus* no)**	2.35 (1.82–3.02)	<0.001
**Country income group (lower *versus* higher)**	1.64 (0.99–2.72)	0.053

CI: confidence interval.

A further analysis was performed only on pwCF with RF/F508del, to compare the characteristics of people carrying the most common class IV or class V variants with the characteristics of pwCF who are homozygous for F508del. To perform this analysis, people without F508del variant (n=1628) and people with rare RF variants (n=953) were excluded.

Table 1 summarises the lists and class of the common specific RF variants in the study group.

### Clinical characteristics of pwCF with class IV variants

A total of 2483 pwCF carried at least one class IV variant. Of these, 1788 pwCF had a compound heterozygous state with F508del and one class IV variant; they were included in the analysis. Notably, their sweat chloride concentrations were significantly lower than those of pwCF who were homozygous for F508del. In addition, their FEV_1_pp across all age groups was significantly higher than that of pwCF homozygous for F508del, and PSA and other airway pathogens were less prevalent in this group (table 2).

To explore the impact of specific class IV variants, we analysed them individually. We compared pwCF who were homozygous for a particular class IV variant with pwCF who were compound heterozygous for F508del and the same class IV variant against pwCF who were homozygous for F508del.

Significant differences were observed among the class IV variants. Notably, the R334W variant was strongly associated with severe lung disease: after adjusting for age, sex, age at diagnosis and country income status, there was no significant difference in FEV_1_pp across all age groups between pwCF carrying F508del/R334W variants and pwCF homozygous for F508del. It's worth noting that 31% of pwCF with F508del/R334W variants were chronically colonised with PSA, compared with 35% of pwCF homozygous for F508del (adjusted p=0.013). There was no significant difference in the colonisation rates of other CF pathogens between the two groups. A small percentage, 4.76%, required oxygen supplementation, and 4.57% had undergone lung transplantation (see supplementary table 4). On the other hand, individuals with other class IV variants (R117H, D1152H, A455E, L206W) displayed significantly better pulmonary function across all age groups, lower rates of PSA colonisation and a reduced prevalence of lung transplantation (supplementary table 4).

### Clinical characteristics of pwCF carrying class V specific variants

At least one class V variant was carried by 2254 pwCF. The most prevalent class V variants were 3849+10 kb C→T, 2789+5G→A and 3272-26A→G. Among these, 1499 pwCF had a compound heterozygous genotype, harbouring F508del and one of the aforementioned class V variants. Notably, their sweat chloride concentration was significantly lower than that of pwCF homozygous for F508del, but significantly higher than pwCF with F508del and class IV variants. Furthermore, across most age groups, their FEV_1_pp was significantly higher than that of pwCF homozygous for F508del. In addition, their FEV_1_pp across all age groups (except 6–11 years) was significantly lower and their PSA colonisation higher than those of pwCF with F508del and class IV variants (table 2).

Significant variations were observed among different class V variants. PwCF carrying the 3849+10 kb C→T variant (and F508del on the other allele) displayed more severe lung disease than pwCF carrying other class V variants (and F508del on the other allele). Despite having lower sweat chloride levels (64 mmol·L^−1^, interquartile range (IQR) 49.2–79) than pwCF homozygous for F508del (100 mmol·L^−1^, IQR, 90–111, adjusted p<0.001), pwCF with the 3849+10 kb C→T variant (and F508del on the other allele) exhibited lung disease that was not significantly different from that of pwCF homozygous for F508del. Their FEV_1_pp across all age groups was similar when adjusted for age, sex, age at diagnosis and country income status. Notably, 38.99% of these pwCF were chronically colonised with PSA, compared with 35.15% of pwCF homozygous for F508del (adjusted p=0.526). There were no differences in the rates of colonisation with other CF pathogens between these two groups (supplementary table 4). 30 pwCF were homozygous for 3849+10 kb C→T. In all parameters, except sweat chloride levels, no significant differences were observed between these pwCF and those who were compound heterozygote for the 3849+10 kb C→T variant (and F508del on the other allele) (supplementary table 4). Interestingly, 31% of pwCF carrying the 3849+10 kb C→T variant were on pancreatic enzymes.

Conversely, other class V variants (2789+5G→A and 3272-26A→G) were associated with significantly better pulmonary function across all age groups, lower rates of PSA colonisation and a reduced prevalence of lung transplantation (supplementary table 4).

PwCF who were homozygous for a specific RF variant were compared with pwCF who were compound heterozygous to the same RF variant and F508del. This comparison was limited to specific RF variants with at least 30 pwCF homozygous for that RF variant.

### Rate of FEV_1_pp decline

A sub-analysis was conducted, involving 15 692 pwCF who had F508del/F508del, F508del/class IV variant or F508del/class V variant genotypes, and who possessed longitudinal FEV_1_pp data spanning a minimum of 3 consecutive years leading up to 2016. Exclusions were made for pwCF aged >50 years due to insufficient data, resulting in a final analysis cohort comprising 15 488 pwCF.

**FIGURE 2:**
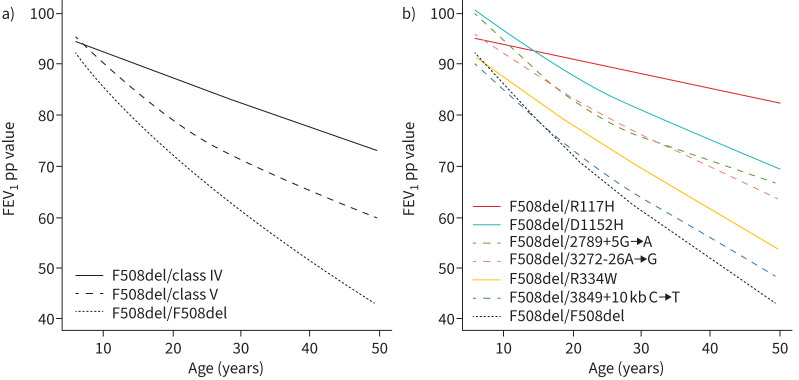
Estimated mean forced expiratory volume in 1 s % predicted (FEV_1_pp) obtained from the mixed-effects model: a) according to age and class IV and class V mutations and F508del and b) according to age and specific residual-function mutations.

**FIGURE 3:**
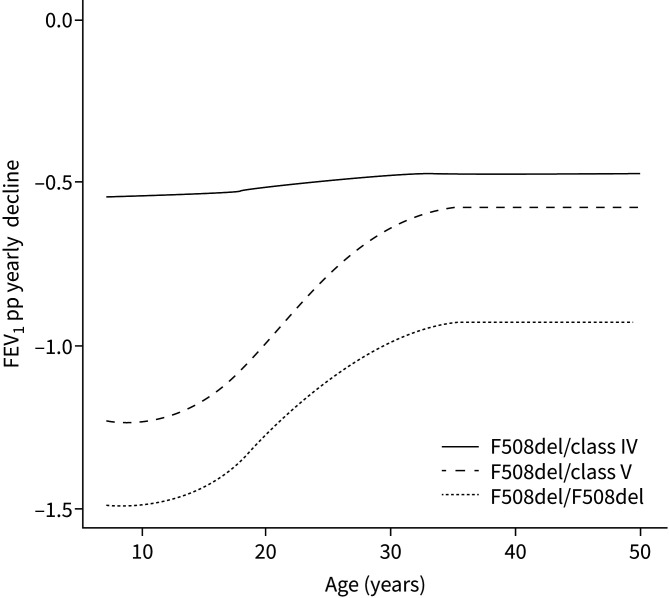
Estimated mean of forced expiratory volume in 1 s % predicted (FEV_1_pp) yearly decline obtained from the mixed-effects model, according to age and mutation group.

Figure 2 illustrates that the rate of FEV_1_pp decline was more pronounced in pwCF carrying class V variants than in pwCF carrying class IV variants. It is important to observe that the decline in FEV_1_pp had already started during childhood for pwCF with class V variants, and the rate of decline was particularly steep during childhood and young adulthood, up to the age of 30, at which point it aligned with the rate observed in pwCF carrying class IV variants (figure 3). Specific variants (3849+10 kb C→T and R334W) exhibited a pattern of FEV_1_pp decline with age akin to that of pwCF with the F508del/F508del genotype.

### Survival analysis

**FIGURE 4:**
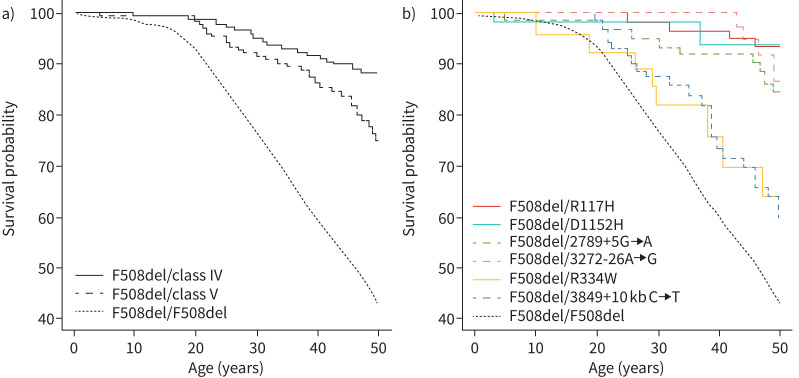
Kaplan–Meier survival curves for people with cystic fibrosis (pwCF) carrying: a) class IV and class V mutations and F508del and b) specific residual-function mutations.

As shown in figure 4, CF significantly shortens the life expectancy of individuals with RF variants, with mortality becoming apparent around the age of 20 years. Furthermore, a Cox regression model was applied, utilising age as the timescale, to assess the influence of variant groups on the likelihood of mortality at a specific age. This analysis considered several adjusting factors, such as sex, age, country income group, chronic PSA infection, CFRD and chronic *Burkholderia cepacia* complex colonisation at the time of entry into the ECFSPR. The Cox model was employed to determine the hazard ratio of death. While the variant group emerged as the most significant variable, residing in lower-income countries, chronic *Burkholderia cepacia* complex infection, female sex and the presence of CFRD were all found to be significantly associated with an elevated risk of mortality (table 3).

### Specific RF variants and pancreatic enzyme supplementation

There was a significant difference in pancreatic enzyme requirements between the different RF variants: while 37% of pwCF carrying the 2789+5G→A variant and 31% of pwCF carrying the 3849+10 kb C→T variant took pancreatic enzymes, only 20% of RF pwCF with the D1152H variant and 18% of pwCF with the R117H variant received enzyme substitution. Only 22.4% of patients homozygous for 2789+5G→A took pancreatic enzymes, compared with 42.14% of pwCF with 2789+5G→A and F508del (supplementary table 4, adjusted p=0.02). Overall, a significantly higher percentage of pwCF carrying class V variants (36.1%) than pwCF carrying class IV variants (22.8%) (p-value<0.001) took pancreatic enzymes.

## Discussion

This study delves into the multifaceted landscape of CF by exploring the clinical outcomes associated with RF variants in the CFTR gene. CF, although classified as a monogenic disorder, exhibits a wide spectrum of disease expressions. To our knowledge, this is the first study to comprehensively assess the characteristics of various RF variants based on an extensive database; within this diverse CF population, we unveiled a remarkable diversity in the clinical presentations associated with these variants. Furthermore, certain RF variants, such as the 3849+10 kb C→T variant, presented a clinical picture comparable to that of patients homozygous for the F508del variant. It is important to note that this comparison was made prior to the widespread availability of elexacaftor/tezacaftor/ivacaftor (ETI), a medication that has significantly improved the outcomes of patients who are homozygous and heterozygous for the F508del variant. As a result, some individuals carrying specific RF variants who are not eligible for ETI might now experience even worse clinical outcomes than patients homozygous for F508del.

In a previous report in which we analysed data from the ECFSPR [6], we revealed that pwCF carrying RF variants exhibit better pulmonary function (FEV_1_pp) across various age groups. However, their FEV_1_pp values decline with age, and a significant proportion of RF patients eventually show FEV_1_pp values comparable to those of patients with MF variants in the same age groups. Notably, individuals with RF variants also have a shorter lifespan than the non-CF population, with mortality becoming apparent around the age of 20 years. Furthermore, >20% had chronic PSA infection, and a substantial portion required intensive daily treatments, including nebulisations and physiotherapy [6]. In this study, we showed that having two RF variants does not result in a better clinical status than having MF/RF variants. Of note, our RF/RF people were younger than the ones carrying MF/RF variants (supplementary table 3), which should have resulted in a milder disease. However, FEV_1_, which is age-specific, was not different between the groups.

Prior studies focusing on specific RF variants were limited by small sample sizes. Sawicki *et al*. [11] had previously described the trajectory of pwCF with F508del/RF variants, indicating a progressive decline in lung function across all age groups. However, our current study underscores that, unlike patients with MF variants for whom studies have failed to establish correlations between specific variants and disease severity, different RF variants within the same class exhibit a wide spectrum of pulmonary disease. This intriguing phenomenon within RF variants is likely to be associated with different molecular mechanisms by which the variant impairs activity. While individuals with MF variants lack functional CFTR, those with RF variants retain some residual CFTR function, which can differ between variants within the same class. In fact, several studies have identified correlations between CFTR activity in RF variants and disease severity. For instance, Hirtz *et al*. demonstrated that anion secretion by rectal epithelial cells correlated with milder disease [12]. Ramalho *et al*. [13], in their examination of residual CFTR function in different RF variants using Forskolin-induced organoid swelling, found varying responses among these variants, which correlated with sweat chloride levels.

A sub-analysis in the current study observed a more pronounced decline in FEV_1_pp among pwCF carrying class V CFTR variants than among those with class IV variants. This could be attributed to several factors and complex interactions within these groups. Class V variants, which are often referred to as splicing variants, typically affect the way the CFTR protein is produced, leading to less functional CFTR protein. As a result, individuals with class V variants may have fewer functioning CFTR channels, contributing to a more rapid decline in lung function. In addition, even within class V variants, there is considerable heterogeneity in terms of the specific variant and its impact on CFTR function. Some class V variants may be associated with slightly higher residual CFTR activity, whereas others, such as the 3849+10 kb C→T variant, may result in very limited activity. However, the residual pancreatic activity of this variant, as well as the lower sweat chloride values, might be due to differences in the CFTR activity in different organs. This clinical heterogeneity can lead to varying disease severity and progression. The differences observed in the sub-analysis may also be influenced by the size of the study group. Therefore, further research is needed to explore the specific mechanisms behind this finding. Understanding this can help inform personalised treatment approaches for individuals with different CFTR variants and contribute to improved outcomes for people living with CF.

Specific class IV variants might contribute to the milder course of this class. The D1152H variant has varying consequences and some people carrying this variant with a CF-causing variant do not have CF. Similarly, the pathogenicity of the R117H variant relies on the poly T tract R117H variant. The variable clinical presentation is probably associated with some CFTR function, as described in CFTR2, while other variants, such as R334W, have only 1.3% activity in the Fischer rate thyroid (FRT) cell line (CFTR2). This led us to analyse other class IV variants – R334W, A455E and L206W – separately. The current study highlights notable distinctions among specific RF variants. For instance, patients carrying the 3849+10 kb C→T variant exhibited a disease severity similar to that of those homozygous for F508del. By contrast, individuals with the D1152H variant presented with a much milder disease. However, it is important to recognise that, even within the D1152H variant, there is considerable heterogeneity, as described by Mussaffi *et al*. [14] and others [15]. In addition, a delayed diagnosis was associated with more severe lung disease, even in cases of variants that were reported to be associated with mild disease.

This report underscores the complex interplay between CFTR variants and the resulting disease severity, emphasising the importance of understanding the nuances of CFTR activity in the context of individual variants. It sheds light on the substantial diversity within RF variants and the potential impact of recent advancements in CF treatments, offering valuable insights for clinical management and personalised care for patients with CF.

The limitations of the classification to classes have been recently raised. Although some variants fall clearly into one class, others lead to several functional defects [16]; however, the classification is broadly used in research and clinical settings.

Disease registries that include thousands of patients from many countries have limitations.

The absence of data may impact diagnostic precision. The pathogenicity of the R117H variant relies on the poly T tract, but since these details are not consistently recorded, its inclusion might lead to an overdiagnosis of CF in individuals with this alteration. Adhering to variable definitions could influence diagnosis, potentially recruiting borderline cases. Newborn screening may identify infants diagnosed as having CF in some countries, but categorised as ‘cystic fibrosis screen positive, inconclusive diagnosis’ (CFSPID) in others, such as those with the R117H (7T/9T) variant on one allele.

Rigour in diagnostic confirmation of CF in patients with RF mutations is important and a robust diagnostic protocol to ensure the accurate identification of CF in all patients analysed should be utilised, including comprehensive genetic testing to identify mutations in the CFTR gene and additional functional assays, such as nasal potential difference and intestinal current measurements, to corroborate the diagnosis. These will minimise diagnostic variability and enhance the validity of a CF diagnosis in these patients.

Issues such as data quality processes, missing data and data entry errors can further complicate matters. Various factors contribute to the diverse presentation of different CF populations. Newborn screening, prevalent in developed countries, might detect very mild cases of CF-related variants in individuals who might not otherwise receive a diagnosis. Conversely, individuals with mild symptoms may avoid clinic visits, resulting in a higher representation of more severe cases.

However, the number of patients in this analysis, including from the large countries (UK, France, Germany and Italy) that make up nearly half of the database, supports the robustness of the data. Recently, a large data quality control project has been initiated throughout Europe with significant improvement of data collection and adherence to variable definition [8].

In summary, this comprehensive study provides valuable insights into the clinical outcomes and disease severity associated with RF CFTR variants. It emphasises the need for personalised approaches in CF management and further research into the impact of specific variants on patient outcomes. The findings contribute to our understanding of CF complexity and the potential for tailored treatments and interventions to improve the lives of individuals with CF. The findings offer valuable insights into the interplay between genotype and phenotype in CF, shedding light on the intricate mechanisms governing disease manifestation. By contrast, MF variants have been associated with more uniform severe CF phenotypes.

## Supplementary material

10.1183/23120541.00587-2024.Supp1 **Please note:** supplementary material is not edited by the Editorial Office, and is uploaded as it has been supplied by the author.
Supplementary material 00587-2024.SUPPLEMENT
