# Supplementary material for: Variability in disease severity among cystic fibrosis patients carrying residual-function variants: data from the European Cystic Fibrosis Society Patient Registry
Source: ERJ Open Res. 2025 Jan 13;11(1):00587-2024. doi: 10.1183/23120541.00587-2024 (PMC11726569; doi:10.1183/23120541.00587-2024)
Supplement: Supplementary file 1 [file 00587-2024.SUPPLEMENT.pdf]

Table 1Supp. List of minimal function mutations- Classes I-II (Class III was excluded)

**1001+2T->G**
**1001+4A->C+993delCTTAA**
**1002-2A->G**
**1006_1007delGA**
**1013delAA**
**1027delG**
**1058delC**
**1078delA**
**1078delT**
**1112delT**
**1119delA**
**1138insG**
**1150delA**
**1154_1155insTC**
**1154insTC**
**1155insTC**
**1156dupTA**
**1157insTA**
**1161delC**
**1161insG**
**1185delTC**
**1199delG**
**1213delT**
**1215delG**
**1221delCT**
**1248+1G->A**
**1248+1G->C**
**1248+1G->T**
**1248insATCAA**
**1249-1G->A**
**1249-27delTA**
**124del23**
**124del23bp**
**1259insA**
**1283delA**
**1288insA**
**1288insTA**
**1291delTT**
**1294del7**
**1307delCACTTCT**
**1309delG**
**1341+1G->A**
**1341+1G->T**
**1342-1G->C**
**1342-2A->C**
**1343delG**
**1354C->T**
**1366delG**

**1367del5**
**1367delC**
**1393-1G->T**
**1429del7**
**1429del7bp**
**1451del5**
**1454insAGAT**
**1460delAT**
**1461ins4**
**1469delT**
**1471delA**
**1497delGG**
**1504delG**
**1516dupA**
**1524+1G->A**
**1525-1G->A**
**1525-2A->G**
**1540del10**
**1543_1555delTATAGTACAG**
**1548delG**
**1550delG**
**1556delT**
**1565delCA**
**1571delG**
**1576insT**
**1597_1601delTCATT**
**1601delT**
**1601delTC**
**1609delCA**
**1612delTT**
**1660delG**
**1677delTA**
**1680-1G->C**
**1706del17**
**1716+1G->A**
**1716+1G->T**
**1716+2T->C**
**1717-1G->A**
**1717-2A->G**
**1717-8G->A**
**1742delAC**
**1749insTA**
**174delA**
**1753delG1766+2T->A**
**175delC**
**175insT**
**1766+2T->A**
**1774delCT**

**1782delA**
**1787delA**
**1802delC**
**1806delA**
**1807delG**
**1811+1.6kbA->G**
**1811+1643G->T**
**1811+1G->A**
**1811+1G->C**
**1811+1G->T**
**1811+2T->C**
**1812-1G->A**
**1812-2A->G**
**1813insC**
**1824delA**
**182delT**
**1833delT**
**1835delT**
**183delC**
**1845delAG/1846delGA**
**185+1G->T**
**186-1G->A**
**186-2A->G**
**1870delG**
**1874insT**
**1898+1G->A**
**1898+1G->C**
**1898+1G->T**
**1898+2T->C**
**1918delGC**
**1924_1930del7**
**1924del17**
**1924del7**
**1932delG**
**1942del17**
**1970delG**
**1972dupA**
**1978delA**
**2003del8**
**2025dupA**
**2033delTC**
**2043delG**
**2051delTT**
**2053insTA**
**2055del9->A**
**2055del9insA**
**2075delA**
**2105_2177del13insAGAAA**

2105-2117del13insAGAAA
2105del13insAGAAA
2108delA
2113delA
2114delT
2118del4
211delG
2132delAC
2141insA
2143delT
2144delT
2176delA
2183AA->G
2183delAA
2184_2185insA
2184delA
2184dupA
2184insA
2185insA
2185insC
2193ins4
21-kbdeletion
2215insG
2289-2295del7bpinsGT
2290del16
2307insA
2335delA
2337delA
2341delC
2347delG
2349dupT
2357dupA
2372del8
237insA
2380_2387del
2406delCC
2409delC
241delAT
2423delG
2429delG
2456delAC
2472_2478del
2481insT
2489_2490insA
2491-1G->C
2493ins8
2512delG
2516delC
2522insC
2554dupT

2556_2557insAT
2556insAT
2566insT
2585delT
2594delGT
2603delT
2619+2T->C
2622+1G->A
2622+1G->T
2622+2T->C
2623-2A->G
2634delT
2634insT
263delTT
2640delT
2686dupT
2694delT
2711delT
2721del11
2723delTT
2732insA
2734G->AT
2742T->G
2745_2746delGT
2747delC
2751+2T->A
2752+2A->T
2752-1G->C
2752-1G->T
2752-2A->G
2766del8
2777insTG
2787del16
2790-1G->C
2790-1G->T
2790-2A->G
2819_2823del5
2819del4bpins13bp
2822delT
2839delA
284delA
2869insG
2896insAG
2907delTT
2908+1G->A
2908+1G->T
2909_2924dup16
2909delT
2921delG
2935del11

2942insT
2948AT->C
2949del5
2954delT
2957delT
295ins8
296+1G->A
296+1G->C
296+1G->T
296+2T->A
296+2T->C
296+2T->G
297-1G->A
297-2A->G
2991del32
2993delT
2998+1G->A
3007delG
300delA
3012delT
3015_3018dupGTCA
3028delA
3029delC
3032T->G
3040+1G->A
3040+2T->C
3040G->C
3041-1G->A
3041delG
3056delGA
306delTAGA
306insA
3079delTT
307dupA
307insA
3100insA
3120+1G->A
3120+2T->C
3120G->A
3121-1G->A
3121-2A->G
3121-2A->T
3121-977_3499+248del2515
3126del4
3130delA
3132delTG
3139+1G->C
3154delG
3171delC
3171insC

3173delAC
317insC
3199del6
3200_3204delTAGTG
3210+1G->A
3231_3232delGT
3238delA
324delC
3271+1G->A
3271+1G->T
3271delG
3271delGG
3272-1G->A
3293delA
3320ins5
3321delG
3349insT
3359delCT
3396delC
3397delC
3419delT
3422del16
3423delC
3425delG
3447delG
3457delA
3468+1G->A
347delC
3495delA
3499+1G->A
3499+1G->T
3499+2T->C
3500-1G->A
3500-2A->G
3500-2A->T
3516del5
3556insAGTA
3577delT
3600+2insT
3600+2T->C
3601-2A->G
360delT
3617delGA
3622insT
3629delT
365-366insT
3659delC
365insT
3667del4
3667ins4

366insC
3670delA
3724delG
3732delA
3737delA
3750delAG
3755delG
3788delC
3789insA
3791delC
3808delG
3821delT
3840delT
3849+1G->A
3850-1G->A
3856delC
3859delC
3859G->T
3860ins31
3876delA
3878delG
3905insT
3906insG
3922del10->C
3940delG
3944delGT
3946delTG
394delTT
3960-3961delA
40_44delAAACT
4005+1G->A
4005+1G->T
4005+2T->C
4010del4
4015delA
4016insT
4021dupT
4022insT
4028delG
4040delA
4048insCC
405+1G->A
405+2T->G
405+3A->C
406-1G->A
406-1G->C
406-1G->T
406-2A->C
406-2A->G
4080delTGTTinsAA

4089ins4
4095+1G->C
4095+1G->T
4095+2T->A
4096-1G->A
4108delT
4114ATA->TT
412del7->TA
4165delGT
4168delCTAAGCC
4171insA
4172delGC
4173delC
4177delG
4203TAG->AA
4209delTGTTinsAA
4209TGTT->AA
4218insT
4225G->T
4243-2A->G
4259del5
4268+2T->C
4271delC
4272delA
4279insA
4301delA
4326delTC
435insA
4374+1G->A
4374+1G->T
4374+2T->C
4375-1G->C
4375-1G->T
4375-2A->C
4375-2A->G
4382delA
441del1A
441delA
4428insGA
442delA
444delA
450delG
451del8
4575+2G->A
457TAT->G
458delAT
460delG
489delC
498delG
519delT

51delC
521+1G->T
525delT
53+2T->C
541del4
541delC
547insGA
547insTA
551_555delTTTCC
552insA
556delA
557delT
565delC
574delA
581_582delGA
583delC
602del14
604_605delTG
605insT
621+1G->A
621+1G->T
621+2T->C
621+2T->G
622-1G->A
622-1G->C
622-2A->C
622-2A->G
624+1G->T
624delT
630delG
63delT
650delATAAA
654del5
657delA
663delT
675del4
681delC
708delT
710_711+5del7
711+1G->T
711+5G->A
712-1G->T
712-2A->G
733delG
749delT
764delT
771-1G->T
840delT
849delG
852del22

870-2A->C
874InsTACA
875+1G->A
875+1G->C
875+2T->C
875delGA
896delT
905delG
908delT
935delA
936delTA
937_938delTC
977insA
982delA
991_995delAACTT
991del5
994del9
A46D
A559T
A561E
c.1977_1987delTTCAATCCT
C225X
C276X
C524X
CFTR40kbdel
CFTR50kbdel
CFTRdele1
CFTRdele1,10
CFTRdele10
CFTRdele10-11
CFTRdele10-24
CFTRdele11
CFTRdele11,12
CFTRdele1-10
CFTRdele1-11
CFTRdele11-16
CFTRdele11-16ins35bp
CFTRdele11-18
CFTRdele11-24
CFTRdele12
CFTRdele12,13
CFTRdele12-14a
CFTRdele1-24
CFTRdele1-26
CFTRdele13
CFTRdele13,14a
CFTRdele13,14b
CFTRdele1-4
CFTRdele14,15
CFTRdele14,15,16,17a,17b

CFTRdele14a
CFTRdele14a-15
CFTRdele14a-17b
CFTRdele14b
CFTRdele14b-15
CFTRdele14b-17b
CFTRdele14b-18
CFTRdele15
CFTRdele15-17
CFTRdele16,17
CFTRdele16,17a
CFTRdele16-17b
CFTRdele16-18
CFTRdele16-20
CFTRdele16-23
CFTRdele17
CFTRdele17,18
CFTRdele17a
CFTRdele17a,17b
CFTRdele17a-18
CFTRdele17b
CFTRdele17b,18
CFTRdele18
CFTRdele18-20
CFTRdele18-21
CFTRdele19
CFTRdele1-9
CFTRdele19,20
CFTRdele19-21
CFTRdele19-22
CFTRdele2
CFTRdele2(ins182)
CFTRdele2(ins186)
CFTRdele2,3
CFTRdele2,3,10
CFTRdele20
CFTRdele20-21
CFTRdele20-23
CFTRdele21
CFTRdele2-10
CFTRdele21-27
CFTRdele2-13
CFTRdele21kb
CFTRdele22
CFTRdele22,23
CFTRdele22-24
CFTRdele22-26
CFTRdele23
CFTRdele24
CFTRdele2-4

CFTRdele25,26
CFTRdele25-27
CFTRdele2-6b
CFTRdele2-8
CFTRdele3
CFTRdele3-10,14b-16
CFTRdele4
CFTRdele4,10
CFTRdele4-10
CFTRdele4-11
CFTRdele4-5
CFTRdele4-6a
CFTRdele4-6ains6bp
CFTRdele4-7
CFTRdele4-7,11-18
CFTRdele4-7,9-10
CFTRdele4-8
CFTRdele4-8,12
CFTRdele4-8,12-21
CFTRdele4ins41bp
CFTRdele5,6
CFTRdele6a-6b
CFTRdele6b
CFTRdele6b-10
CFTRdele7
CFTRdele7-11
CFTRdele7-9
CFTRdele8
CFTRdele8,9
CFTRdele8-10
CFTRdele9
CFTRdelePr-1
CFTRdelePr-3
CFTRdup10-12
CFTRdup10-18
CFTRdup11-13
CFTRdup1-3
CFTRdup14a-19
CFTRdup14b-17b
CFTRdup16,17a
CFTRdup16-18
CFTRdup16-20
CFTRdup16-22
CFTRdup1-6b
CFTRdup17b-18
CFTRdup19
CFTRdup2
CFTRdup22
CFTRdup25-27
CFTRdup3

CFTRdup4-8
CFTRdup6a,6b,8,9
CFTRdup6b,7
CFTRdup6b-10
CFTRdup6b-16
CFTRdup7-11
CFTRdup8-10
D979V
E1046X
E1104X
E1266X
E1308X
E1371X
E1401X
E1418X
E1473X
E193X
E379X
E402X
E479X
E504X
E514X
E54X
E56X
E585X
E60X
E656X
E664X
E692X
E730X
E7X
E815X
E822X
E823X
E826X
E827X
E92K
E92X
F508del
G1003X
G103X
G1061R
G1249R
G1298X
G149X
G194R
G194X
G27R
G27X
G330X

G486X
G542X
G550X
G576X
G628R
G646X
G673X
G745X
G85E
G970R
H1054D
H1375P
H139R
H199Y
I1269N
I1384X
I175V
I507del
K114X
K1177X
K14X
K381X
K442X
K536X
K598X
K688X
K68X
K710X
K716X
K830X
K946X
K978X
L101X
L102R
L1059X
L1065P
L1077P
L1120X
L1243X
L1254X
L1258X
L127X
L1324P
L1335P
L1353X
L159X
L218X
L320X
L453S
L467P

L568X
L719X
L732X
L812X
L867X
L88X
L927P
L941X
M1101K
M1101R
N1303K
p.Ala1067ThrfsX16
p.Ala1136ValfsX7
p.Ala196GlyfsX62
p.Ala554AspfsX14
p.Ala959AspfsX9
p.Arg3GlufsX42
p.Arg764ValfsX11
p.Asn1088LysfsX68
p.Asn1303LysfsX6
p.Asn66LysfsX4
p.Asp1214IlefsX14
p.Asp891AlafsX15
p.Cys1344GlyfsX16
p.Gln1042ThrfsX5
p.Gln237SerfsX21
p.Gln452SerfsX30
p.Gln525AsnfsX2
p.Glu1401GlyfsX61
p.Glu217GlyfsX11
p.Glu407AlafsX4
p.Glu407AsnfsX35
p.Glu528ArgfsX40
p.Glu632ThrfsX9
p.Glu827ArgfsX10
p.Gly1061AspfsX22
p.Gly1123AlafsX13
p.Gly1222ValfsX44
p.Gly1237AlafsX22
p.Gly178TrpfsX5
p.Gly930AspfsX12
p.His856LeufsX40
p.Ile371AsnfsX11
p.Leu1279AlafsX22
p.Leu136HisfsX18
p.Leu1388ProfsX5
p.Leu259SerfsX7
p.Leu435PhefsX7
p.Leu467AsnfsX2
p.Leu578ArgfsX10

p.Leu812TyrfsX10
p.Leu88PhefsX21
p.Leu935AlafsX36
p.Lys1200SerfsX12
p.Lys1461SerfsX7
p.Lys163ArgfsX3
p.Lys381AspfsX5
p.Lys8IlefsX29
p.Met1CysfsX24
p.Met1IlefsX44
p.Phe1111ThrfsX10
p.Phe409LeufsX33
p.Phe490ValfsX36
p.Phe575LeufsX4
p.Ser1347ProfsX13
p.Ser13IlefsX14
p.Ser158LysfsX5
p.Ser549GlufsX9
p.Ser641IlefsX23
p.Ser728TrpfsX5
p.Ser902ThrfsX4
p.Thr1299HisfsX29
p.Thr682GlnfsX40
p.Thr816LeufsX5
p.Thr896IlefsX3
p.Thr940SerfsX34
p.Trp79GlyfsX12
p.Tyr625PhefsX16
p.Tyr89ArgfsX4
p.Val1108CysfsX48
p.Val1240CysfsX4
p.Val1318LeufsX10
p.Val1415AlafsX48
p.Val855IlefsX627
Q1035X
Q1038X
Q1042X
Q1071X
Q1144X
Q1186X
Q1238X
Q1281X
Q1291X
Q1309X
Q1313X
Q1330X
Q1352X
Q1382X
Q1390X
Q1411X

Q1412X
Q1476X
Q151X
Q179X
Q207X
Q220X
Q270X
Q290X
Q2X
Q30X
Q353X
Q359R
Q372X
Q376X
Q378X
Q39X
Q414X
Q493X
Q525X
Q552X
Q634X
Q637X
Q685X
Q689X
Q715X
Q720X
Q744X
Q779X
Q781X
Q799X
Q814X
Q890X
Q98X
Q996X
R1066C
R1102X
R1128X
R1158X
R1162X
R25X
R289X
R347P
R553X
R560K
R560S
R560T
R709X
R75X
R764X
R785X

R792X
R810X
R851X
S1037X
S1159F
S1196X
S1206X
S1248X
S1255X
S1455X
S313X
S434X
S466X
S489X
S4X
S776X
S912X
S962X
T1036N
T388X
V43X
V520F
W1063X
W1089X
W1098C
W1098R
W1098X
W1145X
W1204X
W1274X
W1282X
W1310X
W1316X
W19X
W202X
W216X
W227X
W277X
W356X
W361X
W401X
W496X
W57R
W57X
W679X
W79X
W846X
W882X
Y1092X
Y109X

Y1182X
Y1219X
Y122X
Y1307X
Y1381X
Y1424X
Y161D
Y247X
Y275X
Y304X
Y362X
Y38X
Y517X
Y563D
Y563X
Y569D
Y569X
Y577X
Y849X
Y84X
Y852X
Y913X

Table 2 supp. List of residual function (RF) mutations- Classes IV-V (Number of alleles)

**1898+3A->G (46)**
**2789+5G->A (954)**
**3272-26A->G (471)**
**3849+10kbC->T (918)**
**711+3A->G (66)**
**A1006E (34)**
**A1067T (2)**
**A455E (287)**
**A613T (14)**
**D110E (12)**
**D110H (71)**
**D1152H (529)**
**D513G (6)**
**D579G (60)**
**E193K (7)**
**E474K (5)**
**E56K (3)**
**E831X (43)**
**F311L (8)**
**G126D (20)**
**G91R (6)**
**G970D (10)**
**H609R (19)**
**I1366N (7)**
**I336K (63)**
**I502T (18)**
**I601F (14)**
**L138ins (79)**
**L15P (4)**
**L165S (26)**
**L206W (271)**
**L346P (1)**
**P205S (32)**
**P574H (11)**
**P67L (121)**
**P99L (5)**
**Q98R (5)**
**R1066H (91)**
**R1070Q (31)**
**R117C (133)**
**R117H (1054)**
**R117P (4)**
**R1283M (6)**
**R334L (13)**

Clinical characteristics of the RF group:

Median age of pwCF carrying RF mutations was 24.7 years (IQR- 11 – 40.1). Age of diagnosis 6.5 years (IQR-0.3 - 21.9). Their sweat chloride was 73 mmol\L at diagnosis (IQR- 52.7 - 92.3). 13.7% of pwCF had sweat chloride below 40 mmol\L, 19.7% had sweat chloride 40-60 mmol\L, and 66.6% had sweat chloride above 60 mmol/L. 42.4% of RF pwCF are chronically colonized with at least one typical CF pathogen in their airways. 4.6% are requiring oxygen therapy and 2.7% had lung transplant.

5868 pwCF carry RF/MF mutations while 324 are having RF/RF mutation. RF/RF pwCF are younger than RF/MF pwCF and although they tend to exhibit several parameters suggesting a milder disease, only few of these parameters reach statistical significance, probably due to the small number of RF/RF pwCF (table).

Table 3 suppl. Demographic and clinical characteristics of RF groups according to the other mutation type.

	Residual function N=6192	RF/MF N=5868	RF/RF N=324	p-value*
**Demographic characteristics**				
Age, median (IQR)	24.7 (11.0 - 40.1)	24.9 (11.1 - 40.2)	19.5 (8.9 – 38.1)	0.015
Female	3066 (49.52%)	2906 (49.52%)	160 (49.38%)	1.000
Country low income	672 (10.85%)	629 (10.72%)	43 (13.27%)	1.000
Age at diagnosis, median (IQR)	6.5 (0.3 - 21.9)	6.7 (0.3 - 21.6)	5.1 (0.3 - 24.4)	0.001
**Sputum pathogens**				
Chronic *Pseudomonas Aeruginosa*	1254 (22.27%)	1200 (22.5%)	54 (18.06%)	0.163
Chronic *Burkholderia Cepacia Complex*	98 (1.73%)	97 (1.8%)	1 (0.34%)	0.091
Chronic *Staphylococcus aureus*	1535 (35.75%)	1454 (35.85%)	81 (34.03%)	0.538
At least one chronic infection	2415 (42.41%)	2301 (42.65%)	114 (38.13%)	0.204
Nontuberculous mycobacteria	133 (2.59%)	129 (2.65%)	4 (1.50%)	0.290
Stenotrophomonas maltophilia	306 (5.42%)	296 (5.53%)	10 (3.34%)	0.143
**Complication**				
ABPA	217 (3.67%)	207 (3.69%)	10 (3.25%)	0.902
CF related diabetes	220 (3.72%)	205 (3.66%)	15 (4.85%)	0.092
Haemoptysis major over 250 ml	132 (2.24%)	126 (2.25%)	6 (1.93%)	0.723
Pneumothorax requiring chest drain	18 (0.30%)	17 (0.30%)	1 (0.32%)	0.944
**Treatment**				
Continuous inhaled hypertonic NaCl	1692 (29.66%)	1616 (29.88%)	76 (25.59%)	0.085
Continuous use of rhDNase	2198 (36.32%)	2102 (36.65%)	96 (30.28%)	0.012
Inhaled continuous antibiotic	1661 (27.67%)	1591 (27.98%)	70 (22.15%)	0.073
Continuous azithromycin	1368 (24.11%)	1306 (24.28%)	62 (20.88%)	0.405
Continuous inhaled bronchodilators	3087 (54.06%)	2933 (54.17%)	154 (52.03%)	0.809
Oxygen therapy	275 (4.62%)	263 (4.67%)	12 (3.82%)	0.636
Lung transplantation	166 (2.70%)	159 (2.73%)	7 (2.18%)	0.725
FEV_1pp, median (IQR)				
6-11 years	99.1 (90.6 - 108.0)	99.1 (90.9 - 108.0)	95.9 (86.5 - 107.4)	0.054
- 12-17 years	93.6 (82.0 - 102.4)	93.6 (82.0 - 102.5)	92.7 (81.7 - 99.8)	0.513
- 18-34 years	82.6 (62.5 - 96.0)	82.4 (62.4 - 95.9)	86.1 (63.8 - 98.4)	0.443
- 35-49 years	72.6 (53.3 - 89.6)	72.1 (52.7 - 89.3)	82.5 (60.9 - 96.0)	0.015
- 50+ years	64.8 (46.0 - 85.3)	64.4 (45.6 - 84.8)	79.5 (55.8 - 90.9)	0.074

* p-value was adjusted to age, sex, age at diagnosis, and socioeconomic status

Table 4 suppl. Clinical Characteristics of people with CF homozygous to F508del or carrying F508del and a specific residual function mutation.

	F508del - F508del	F508del - R117H	vs. F508del - F508del p-value*	F508del - 2789+5G->A	vs. F508del - F508del p-value*	F508del - 3849+10kbC->T	vs. F508del - F508del p-value*
**Demographic characteristics**							
Age, median (IQR)	19.6 (10.5 - 29.2)	20.2 (7.9 - 39.3)	0.003	28.3 (14.2 - 42.7)	<0.001	25.7 (16.5 - 36.6)	<0.001
Female	10867 (47.73%)	400 (48.08%)	0.860	295 (52.49%)	0.026	282 (47.96%)	0.933
Country low income	2466 (10.83%)	15 (1.8%)	<0.001	53 (9.43%)	0.335	138 (23.47%)	<0.001
Age at diagnosis, median (IQR)	0.2 (0.1 - 1.4)	2.6 (0.1 - 24.7)	<0.001	7.8 (0.2 - 22.9)	<0.001	10.2 (4 - 17.6)	0.001
Sweat Chloride, median (IQR)	100 (90 - 111)	46.4 (32 - 66)	<0.001	96 (83 - 109)	<0.001	64 (49.2 - 79)	<0.001
BMI z-score, median (IQR)	-0.4 (-1.2 - 0.2)	0.5 (-0.3 - 1.1)	<0.001	0 (-0.7 - 0.8)	<0.001	-0.2 (-1 - 0.5)	<0.001
**Sputum pathogens**							
Chronic *Pseudomonas Aeruginosa*	6716 (35.13%)	63 (8.38%)	<0.001	138 (27.99%)	<0.001	193 (38.99%)	0.526
Chronic *Burkholderia Cepacia Complex*	728 (3.8%)	8 (1.03%)	0.001	11 (2.22%)	0.072	18 (3.64%)	0.760
Chronic *Staphylococcus aureus*	5459 (36.45%)	77 (13.3%)	<0.001	168 (49.41%)	<0.001	169 (42.14%)	0.853
At least one chronic infection	10668 (55.46%)	130 (16.69%)	<0.001	262 (52.72%)	<0.001	319 (64.19%)	0.446
Nontuberculous mycobacteria	670 (3.98%)	4 (0.55%)	<0.001	5 (1.12%)	0.001	20 (4.83%)	0.590
Stenotrophomonas maltophilia	1731 (9.04%)	23 (3.03%)	<0.001	29 (5.84%)	0.022	35 (7.04%)	0.509
**Complication**							
ABPA	1487 (6.89%)	17 (2.14%)	<0.001	20 (3.75%)	0.033	21 (3.82%)	0.247
CF related diabetes	4897 (22.33%)	26 (3.24%)	<0.001	28 (5.26%)	<0.001	17 (3.09%)	<0.001
Haemoptysis major over 250 ml	576 (2.72%)	4 (0.51%)	0.002	12 (2.22%)	0.147	23 (4.27%)	0.153
Pneumothorax requiring chest drain	198 (0.91%)	0 (0%)	0.968	0 (0%)	0.959	4 (0.72%)	0.276
**Treatment**							
Continuous inhaled hypertonic NaCl	8256 (43.57%)	100 (12.9%)	<0.001	146 (28.85%)	<0.001	216 (43.81%)	0.510
Continuous use of rhDNase	13048 (59.2%)	168 (20.54%)	<0.001	208 (38.1%)	<0.001	338 (59.72%)	0.593
Inhaled continuous antibiotic	11015 (51.05%)	105 (13.03%)	<0.001	156 (28.68%)	<0.001	276 (50.09%)	0.472
Continuous azithromycin	7499 (39.75%)	114 (14.77%)	<0.001	132 (26.04%)	<0.001	180 (36.73%)	0.188
Continuous inhaled bronchodilators	12500 (65.97%)	256 (33.03%)	<0.001	286 (56.63%)	<0.001	359 (72.53%)	0.008
Oxygen therapy	1857 (8.58%)	25 (3.18%)	<0.001	23 (4.26%)	<0.001	54 (9.64%)	0.752
Pancreatic enzymes	21565 (97.8%)	139 (17.62%)	<0.001	228 (42.14%)	<0.001	189 (33.87%)	<0.001
Ursodeoxycholic acid	8036 (36.48%)	33 (4.03%)	<0.001	80 (14.73%)	<0.001	134 (23.76%)	<0.001
Lung transplantation	1742 (7.78%)	9 (1.09%)	<0.001	24 (4.3%)	<0.001	38 (6.59%)	0.038
FEV1 % predicted, median (IQR)							
- 6-11 years	94.5 (82.7 - 103.7)	97.9 (89.8 - 108.3)	<0.001	103.7 (97.1 - 111.9)	<0.001	95.5 (84.2 - 105.6)	0.761
- 12-17 years	83.1 (68.4 - 94.9)	93.1 (85.8 - 99.4)	<0.001	95.9 (84.8 - 102.4)	<0.001	83.4 (61.7 - 95.6)	0.347
- 18-34 years	66 (45.5 - 83.1)	93.4 (82 - 101.1)	<0.001	82 (62.7 - 96)	<0.001	68.7 (49.5 - 83.7)	0.681
- 35-49 years	55 (38 - 75.7)	90.5 (75.6 - 102)	<0.001	72.6 (54.6 - 88.9)	<0.001	52.9 (38.3 - 70.9)	0.055

- 50+ years	51.8 (34.2 - 71.3)	67.6 (48.5 - 87.4)	<0.001	59.2 (38.6 - 77.9)	0.054	53 (40.1 - 69.4)	0.703

	F508del - D1152H	vs. F508del - F508del p-value*	F508del - 3272-26A->G	vs. F508del - F508del p-value*	F508del - R334W	vs. F508del - F508del p-value*	F508del - A455E	vs. F508del - F508del p-value*
**Demographic characteristics**								
Age, median (IQR)	31.5 (10 - 47.5)	<0.001	30.5 (15.4 - 44.5)	<0.001	24.9 (11.9 - 36.9)	<0.001	30.9 (17.5 - 44.4)	<0.001
Female	175 (54.18%)	0.022	160 (45.85%)	0.517	102 (46.58%)	0.786	122 (53.28%)	0.097
Country low income	17 (5.26%)	0.001	39 (11.17%)	0.795	48 (21.92%)	<0.001	1 (0.44%)	<0.001
Age at diagnosis, median (IQR)	22.2 (0.4 - 36)	<0.001	8.2 (0.5 - 21)	<0.001	5.9 (0.4 - 17.1)	<0.001	12 (0.8 - 22.6)	<0.001
Sweat Chloride, median (IQR)	40 (30 - 57.5)	<0.001	90.5 (79.8 - 102.2)	<0.001	93.2 (85 - 109)	0.001	75 (64 - 91.2)	<0.001
BMI z-score, median (IQR)	0.3 (-0.4 - 1.1)	<0.001	0.2 (-0.6 - 0.9)	<0.001	0 (-0.7 - 0.7)	<0.001	0.3 (-0.4 - 1)	<0.001
**Sputum pathogens**								
Chronic *Pseudomonas Aeruginosa*	55 (18.39%)	<0.001	80 (25.56%)	<0.001	62 (31.31%)	0.013	55 (26.7%)	<0.001
Chronic *Burkholderia Cepacia Complex*	2 (0.66%)	0.039	8 (2.54%)	0.139	7 (3.52%)	0.516	3 (1.45%)	0.090
Chronic *Staphylococcus aureus*	66 (28.82%)	0.002	87 (40.28%)	0.919	75 (46.58%)	0.207	50 (33.78%)	0.309
At least one chronic infection	109 (36.09%)	<0.001	146 (46.35%)	<0.001	110 (55%)	0.058	97 (46.86%)	<0.001
Nontuberculous mycobacteria	9 (3.11%)	0.106	10 (3.62%)	0.333	5 (2.98%)	0.459	7 (3.37%)	0.230
Stenotrophomonas maltophilia	16 (5.32%)	0.034	30 (9.49%)	0.591	11 (5.58%)	0.193	20 (9.39%)	0.897
**Complication**								
ABPA	11 (3.58%)	0.165	22 (6.67%)	0.568	7 (3.35%)	0.206	14 (6.51%)	0.813
CF related diabetes	8 (2.61%)	<0.001	14 (4.26%)	<0.001	5 (2.45%)	<0.001	16 (7.24%)	<0.001
Haemoptysis major over 250 ml	9 (2.92%)	0.888	9 (2.72%)	0.409	9 (4.37%)	0.245	8 (3.65%)	0.666
Pneumothorax requiring chest drain	2 (0.64%)	0.226	1 (0.3%)	0.158	1 (0.48%)	0.343	0 (0%)	0.961
**Treatment**								
Continuous inhaled hypertonic NaCl	77 (25.25%)	<0.001	109 (34.28%)	0.003	81 (40.7%)	0.145	60 (27.65%)	<0.001
Continuous use of rhDNase	86 (27.39%)	<0.001	169 (49.71%)	0.015	97 (45.33%)	<0.001	113 (50.67%)	0.540
Inhaled continuous antibiotic	75 (24.04%)	<0.001	123 (36.28%)	<0.001	86 (40.95%)	0.003	70 (31.67%)	<0.001
Continuous azithromycin	60 (19.74%)	<0.001	114 (36.08%)	0.001	52 (26.26%)	<0.001	73 (33.18%)	<0.001
Continuous inhaled bronchodilators	159 (52.13%)	<0.001	198 (62.07%)	0.031	133 (66.83%)	0.893	111 (50.45%)	<0.001
Oxygen therapy	15 (4.92%)	0.003	15 (4.5%)	<0.001	10 (4.76%)	0.027	9 (4.05%)	0.001
Pancreatic enzymes	70 (22.65%)	<0.001	100 (30.03%)	<0.001	88 (41.51%)	<0.001	67 (30.04%)	<0.001
Ursodeoxycholic acid	30 (9.55%)	<0.001	26 (7.76%)	<0.001	30 (14.08%)	<0.001	16 (7.17%)	<0.001
Lung transplantation	6 (1.9%)	<0.001	10 (2.9%)	<0.001	10 (4.57%)	0.037	10 (4.41%)	<0.001
FEV1 % predicted, median (IQR)								
- 6-11 years	100.9 (94.3 - 106.9)	0.006	96.4 (89 - 104.4)	0.224	99.1 (95.3 - 107.7)	0.142	99.7 (90.7 - 104.1)	0.298
- 12-17 years	100.6 (91 - 116.6)	0.001	96 (82.9 - 101.6)	0.004	85.4 (78.1 - 98.8)	0.291	92.4 (81.1 - 100.7)	0.154
- 18-34 years	87.6 (71.1 - 94.7)	<0.001	81.7 (66 - 95.8)	<0.001	71 (47 - 82.8)	0.449	78.1 (62.9 - 89.1)	0.010
- 35-49 years	75 (58.7 - 87.2)	0.023	69.9 (46.8 - 86.1)	0.018	72.9 (49.6 - 87.8)	0.055	64.8 (55.6 - 82.8)	0.134
- 50+ years	69.5 (47.5 - 92.1)	0.001	62.8 (42.9 - 80.3)	0.078	63.3 (43.6 - 73.7)	0.488	72.7 (63.1 - 97.6)	<0.001

	F508del - L206W	vs. F508del - F508del p-value*	2789+5G->A - 2789+5G->A	vs. F508del - 2789+5G->A p-value*	vs. F508del - F508del p-value*	3849+10kbC->T - 3849+10kbC->T	vs. F508del - 3849+10kbC->T p-value*	vs. F508del - F508del p-value*
**Demographic characteristics**								
Age, median (IQR)	18.5 (7.3 - 38.3)	0.683	17.6 (9.7 - 35.5)	0.022	0.905	27.8 (17.4 - 39.9)	0.799	0.017
Female	87 (47.03%)	0.883	18 (40%)	0.122	0.370	17 (56.67%)	0.454	0.364
Country low income	2 (1.08%)	<0.001	9 (20%)	0.037	0.055	10 (33.33%)	0.271	0.001
Age at diagnosis, median (IQR)	0.9 (0.2 - 25.5)	<0.001	4.5 (0.4 - 13.4)	0.569	<0.001	17.3 (5.9 - 24.1)	0.036	<0.001
Sweat Chloride, median (IQR)	64.5 (54 - 78.2)	<0.001	85 (75 - 100)	0.050	<0.001	52 (32 - 62.2)	0.036	<0.001
BMI z-score, median (IQR)	0.6 (-0.5 - 1.3)	<0.001	0.3 (-0.7 - 1)	0.386	0.001	0 (-0.6 - 0.5)	0.518	0.192
**Sputum pathogens**								
Chronic *Pseudomonas Aeruginosa*	8 (4.57%)	<0.001	5 (12.5%)	0.099	0.001	12 (42.86%)	0.618	0.714
Chronic *Burkholderia Cepacia Complex*	1 (0.57%)	0.112	0 (0%)	0.992	0.957	1 (3.57%)	0.901	0.866
Chronic *Staphylococcus aureus*	20 (20.62%)	0.001	5 (13.51%)	<0.001	0.002	12 (52.17%)	0.631	0.798
At least one chronic infection	27 (15.34%)	<0.001	10 (25%)	0.003	<0.001	19 (67.86%)	0.957	0.828
Nontuberculous mycobacteria	4 (2.47%)	0.165	0 (0%)	0.996	0.960	0 (0%)	0.991	0.949
Stenotrophomonas maltophilia	6 (3.43%)	0.014	0 (0%)	0.988	0.930	4 (14.29%)	0.271	0.145
**Complication**								
ABPA	3 (1.65%)	0.032	0 (0%)	0.992	0.932	3 (10.71%)	0.064	0.065
CF related diabetes	5 (2.76%)	<0.001	6 (15%)	0.001	0.329	1 (3.57%)	0.749	0.028
Haemoptysis major over 250 ml	0 (0%)	0.942	0 (0%)	0.992	0.957	3 (10.71%)	0.184	0.038
Pneumothorax requiring chest drain	1 (0.55%)	0.613	1 (2.38%)	0.996	0.467	0 (0%)	0.994	0.966
**Treatment**								
Continuous inhaled hypertonic NaCl	30 (17.05%)	<0.001	6 (14.63%)	0.061	<0.001	8 (29.63%)	0.170	0.076
Continuous use of rhDNase	33 (18.13%)	<0.001	14 (31.82%)	0.233	0.001	13 (48.15%)	0.136	0.327
Inhaled continuous antibiotic	19 (10.44%)	<0.001	6 (13.95%)	0.113	<0.001	15 (53.57%)	0.885	0.751
Continuous azithromycin	27 (15.34%)	<0.001	6 (14.63%)	0.191	0.002	11 (40.74%)	0.409	0.533
Continuous inhaled bronchodilators	62 (35.23%)	<0.001	23 (56.1%)	0.558	0.234	15 (55.56%)	0.093	0.419
Oxygen therapy	3 (1.69%)	0.006	2 (4.55%)	0.825	0.302	2 (7.41%)	0.607	0.675
Pancreatic enzymes	27 (15%)	<0.001	10 (22.73%)	0.018	<0.001	7 (25.93%)	0.142	<0.001
Ursodeoxycholic acid	12 (6.59%)	<0.001	9 (20.45%)	0.461	0.009	6 (22.22%)	0.549	0.021
Lung transplantation	0 (0%)	0.931	1 (2.22%)	0.772	0.124	0 (0%)	0.988	0.936
FEV1 % predicted, median (IQR)								
- 6-11 years	103.6 (94.9 - 113.3)	0.003	103.7 (96 -	0.478	0.500	71 (60.6 -	0.021	0.044

				111.3)			81.4)		
-	12-17 years	100.6 (94.3 - 105.1)	<0.001	101.5 (95.8 - 107.7)	0.053	0.002	76.2 (57.8 - 76.6)	0.528	0.250
-	18-34 years	94.4 (86.9 - 98.5)	<0.001	101 (92.1 - 106.8)	0.146	<0.001	65.9 (41.7 - 75.8)	0.703	0.443
-	35-49 years	81.4 (63.1 - 98.9)	<0.001	92.8 (88.8 - 99)	0.067	0.029	51.3 (27.5 - 70.1)	0.608	0.193
-	50+ years	71.4 (54.6 - 90.1)	0.012	68.8 (45.8 - 87.6)	0.948	0.419	52.6 (52.6 - 52.6)	0.883	0.931

* Except for demographic characteristics, p-value was adjusted to age, sex, age at diagnosis, and socioeconomic status.
